# Supplementary material for: MetaRibo-Seq measures translation in microbiomes
Source: Nat Commun. 2020 Jun 29;11:3268. doi: 10.1038/s41467-020-17081-z (PMC7324362; doi:10.1038/s41467-020-17081-z)
Supplement: Supplementary file 10 — Supplementary Data 7 [file 41467_2020_17081_MOESM10_ESM.zip › File2/Confidence_High_Taxonomy/85379_out.krona.html]

Javascript must be enabled to view this page.

members
magnitude
magnitudeUnassigned
count
unassigned
taxon
rank

85379\_out

49

2
49
superkingdom

49
phylum
976

200643
49
class

49
order
171549

family
49
171550

239759
genus
49

SRS146812\_contig\_number\_contig-100\_2395.323787
1


SRS078419\_contig\_number\_9426SRS143991\_contig\_number\_22901
2
1118061
species


SRS015782\_contig\_number\_contig-100\_15508.149906SRS019808\_contig\_number\_contig-100\_1655.82115SRS024132\_contig\_number\_32716SRS051882\_contig\_number\_contig-100\_14863.86372SRS052697\_contig\_number\_contig-100\_14206.148227SRS1055022\_contig\_number\_contig-100\_11769.49446SRS1055069\_contig\_number\_16556SRS148970\_contig\_number\_contig-100\_1214.40736
8
1262694
species

37
species
214856

SRS013965\_contig\_number\_38482SRS014979\_contig\_number\_28770SRS016056\_contig\_number\_2754SRS017247\_contig\_number\_contig-100\_1574.53899SRS018351\_contig\_number\_12816SRS018984\_contig\_number\_20126SRS019068\_contig\_number\_63863SRS019161\_contig\_number\_contig-100\_2424.212803SRS020394\_contig\_number\_17028SRS024075\_contig\_number\_13562SRS043411\_contig\_number\_17113SRS043701\_contig\_number\_16598SRS051610\_contig\_number\_4446SRS057480\_contig\_number\_9600SRS064276\_contig\_number\_5153SRS064645\_contig\_number\_35SRS075984\_contig\_number\_18896SRS077127\_contig\_number\_10663SRS077335\_contig\_number\_2554SRS077454\_contig\_number\_6110SRS077552\_contig\_number\_22418SRS097920\_contig\_number\_18652SRS098827\_contig\_number\_9193SRS1041142\_contig\_number\_contig-100\_1164.56476SRS1041145\_contig\_number\_38379SRS1041164\_contig\_number\_contig-100\_4819.31628SRS1055076\_contig\_number\_14013SRS1055083\_contig\_number\_13262SRS142542\_contig\_number\_1212SRS143466\_contig\_number\_29118SRS143722\_contig\_number\_15944SRS144183\_contig\_number\_40976SRS146813\_contig\_number\_4863SRS147039\_contig\_number\_844SRS147653\_contig\_number\_3195SRS148253\_contig\_number\_contig-100\_630.79020SRS148817\_contig\_number\_contig-100\_914.73612


SRS143342\_contig\_number\_21556
1
328813
species
